# Supplementary material for: SWI/SNF chromatin remodeler complex within the reward pathway is required for behavioral adaptations to stress
Source: Nat Commun. 2022 Apr 4;13:1807. doi: 10.1038/s41467-022-29380-8 (PMC8980038; doi:10.1038/s41467-022-29380-8)
Supplement: Supplementary file 1 — Supplementary Information [file 41467_2022_29380_MOESM1_ESM.pdf]

SWI/SNF chromatin remodeler complex within the reward pathway is required for behavioral adaptations to stress

Abdallah Zayed[1], Camille Baranowski[1], Anne-Claire Compagnion[1], Cécile Vernochet[1], Samah Karaki[1], Romain Durand-de Cuttoli[2], Estefani Saint-Jour[3], Soumee Bhattacharya[1], Fabio Marti[2,5], Peter Vanhoutte[3], Moshe Yaniv[4], Philippe Faure[2,5], Jacques Barik[1,6,7], Laurence Amar[1], François Tronche[1]* and Sébastien Parnaudeau[1]*.

1. Sorbonne Université, Gene Regulation and Adaptive Behaviors, Neuroscience Paris-Seine, IBPS. CNRS UMR8246, INSERM, Paris, France
2. Sorbonne Université, Neurophysiology and Behaviors, Neuroscience Paris-Seine, IBPS. CNRS UMR8246, INSERM, UPMC, Paris, France
3. Sorbonne Université, Neuronal Signaling and Gene Regulation, Neuroscience Paris-Seine, IBPS. CNRS UMR8246, INSERM, UPMC, Paris, France
4. Developmental and Stem Cell Biology department, Pasteur Institute, Paris, France
5. Present address, Brain Plasticity Unit, CNRS, ESPCI Paris, PSL Research University, Paris, France.
6. Present address, Université Côte d'Azur, Nice, France.
7. Present address, Physiopathology of Neuronal Circuits and Behavior, IPMC, CNRS UMR7275, Valbonne, France
* These authors contributed equally
Corresponding author: François Tronche
Email: francois.tronche@sorbonne-universite.fr

Supplementary figures S1 to S12

Supplementary tables S1 to S3

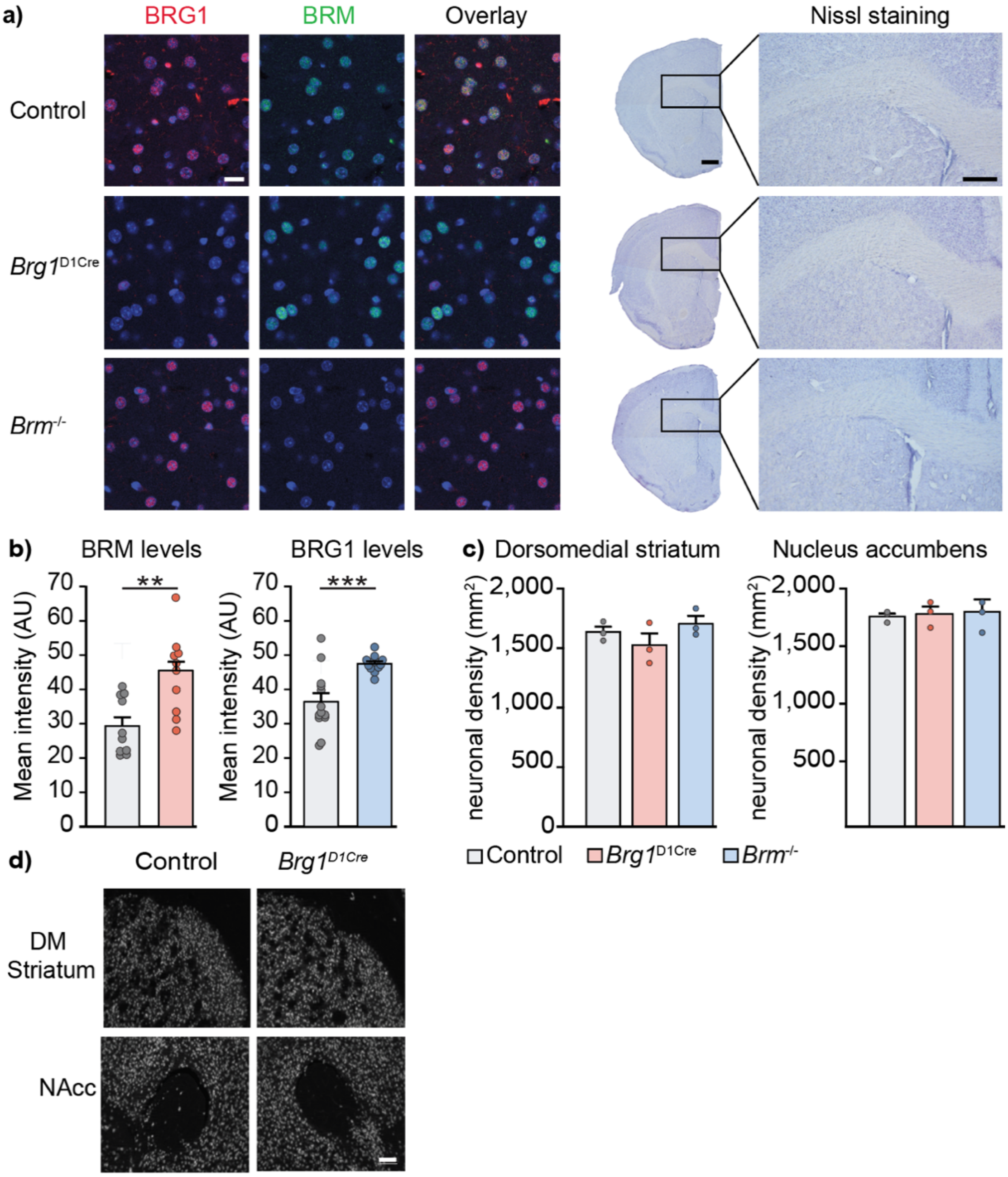

Supplementary Figure 1: Genetic inactivation of *Brg1* in dopaminoceptive neurons in *Brg1*^{D1Cre} mice and of *Brm* in the whole organism in *Brm*$^{-/-}$ mice.

(a) Left panel: BRG1 (red) and BRM (green) co-staining within the nucleus accumbens of control (upper row), *Brg1*^{D1Cre} (middle row), and *Brm*$^{-/-}$ (lower row) mice. DAPI nuclear staining is in blue. Scale bar = 25 µm. Right panel: Nissl staining from striatal coronal slices of control (upper row), *Brg1*^{D1Cre} (middle row), and *Brm*$^{-/-}$ (lower row). Scale bar=200 µm. **(b)** Left panel: BRM expression levels in control (n=10 cells in two mice) and *Brg1*^{D1Cre} mice (n=10 in two mice) striatal neurons. Two-sided t-test, **p=0.0036. Right panel: BRG1 expression levels in control (n=13 cells in two mice) and *Brm*$^{-/-}$ mice (n=12 in two mice) striatal neurons. Two-sided t-test, ***p=0.0003. **(c)** Neuronal density using NeuN staining in the dorsomedial striatum and nucleus accumbens of control (n=3), *Brg1*^{D1Cre} (n=3) and *Brm*$^{-/-}$ mice (n=3). **(d)** Representative image of NeuN staining in the dorsomedial striatum and nucleus accumbens. Scale bar = 100 µm. Data are represented as mean ± s.e.m. Data are provided in a Source Data file.

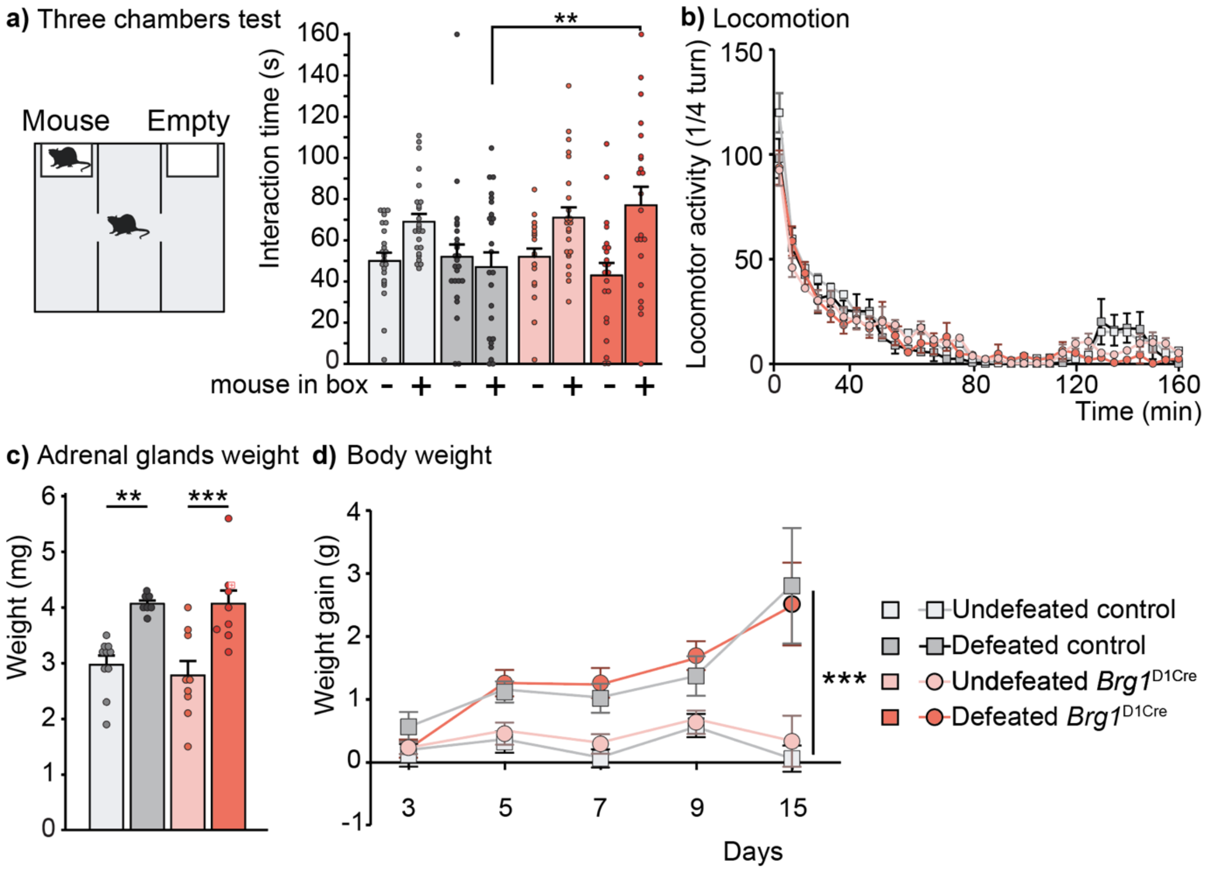

Supplementary Figure 2: Behavioral and anatomical consequences of repeated social defeat in controls and *Brg1*^{D1Cre}.

(a) Left panel: schematic view of the three chambers interaction test. Right panel: social interaction in the three chambers test. Undefeated control n=25, defeated control n=24, undefeated *Brg1*^{D1Cre} n=24, defeated *Brg1*^{D1Cre} n=22. Main genotype effect F(1,91)=5.1; p=0.0264. Main stress effect F(1,91)=4.32; p=0.0405. Main social target effect F(1,91)=11.9; p=0.0009. Three-way ANOVA with repeated measure followed by Bonferroni correction **p=0.051. **(b)** Effect of repeated social defeat on locomotor activity in control and *Brg1*^{D1Cre} mice. Undefeated control n=9, defeated control n=6, undefeated *Brg1*^{D1Cre} n=8, defeated *Brg1*^{D1Cre} n=9. **(c)** Adrenal glands weight in undefeated control n=10, defeated control n=7, undefeated *Brg1*^{D1Cre} n=9, defeated *Brg1*^{D1Cre} n=9. Main stress effect F(1,31)=32.8; p<0.0001. Two-way ANOVA followed by Bonferroni correction control undefeated *vs* control defeated **p=0.0019; *Brg1*^{D1Cre} undefeated *vs* *Brg1*^{D1Cre} defeated ***p=0.0002. **(d)** Body weight gain from the day prior repeated social defeat in undefeated control n=12, defeated control n=12, undefeated *Brg1*^{D1Cre} n=13, defeated *Brg1*^{D1Cre} n=13. Three-way ANOVA with repeated measure, interaction stress x days F(4,184)=10.37; ***p<0.0001. Data are represented as mean ± s.e.m. Data are provided in a Source Data file.

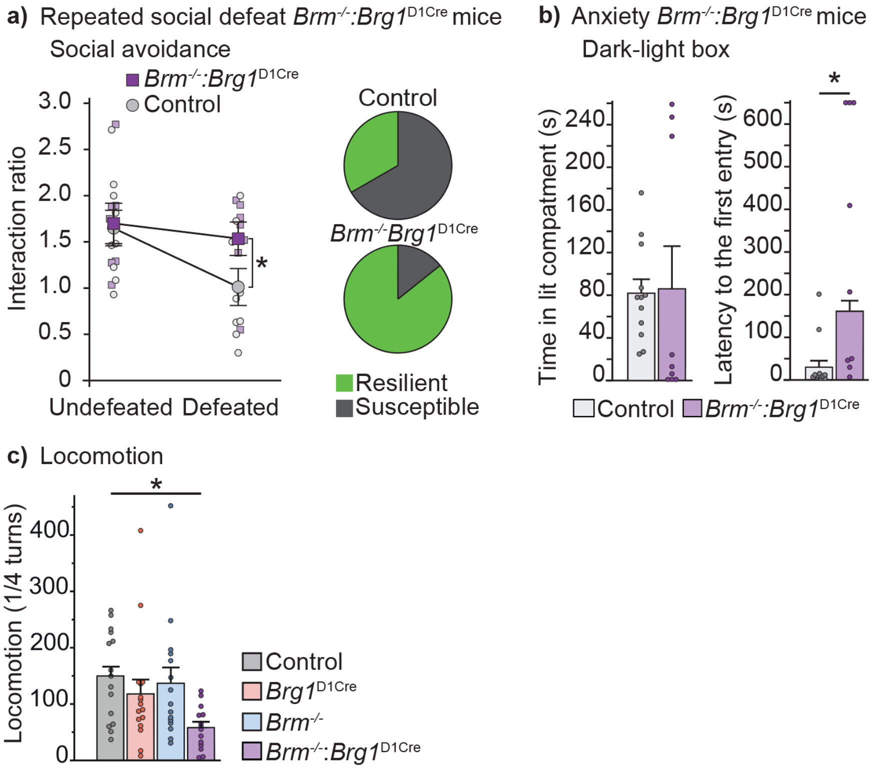

Supplementary Figure 3: Behavioral consequences of repeated social defeat in controls and compound mutants.

(a) Effect of repeated social defeat on social interaction time in control and *Brg1*^{D1Cre}:*Brm*$^{-/-}$ mice (left panel). Note that the same mice have been tested before and after repeated social defeat. Hence the data are represented in terms of ratio between the interaction time with a mouse and the time spent in the vicinity of the box while empty. n=9 (control mice); n=7 (compound mutant mice). Main stress effect F(1,14)=5.09; p<0.0405. Two-way ANOVA with repeated measure followed by Bonferroni correction control unstressed *vs* control stressed *p=0.0347. Right panel, percentage of resilient (in green) and susceptible mice (in gray) in defeated controls (n=9) and defeated *Brg1*^{D1Cre}:*Brm*$^{-/-}$ mice (n=7). X^2(1,16)=4.39; p=0.0361. **(b)** Anxiety-like behavior of *Brg1*^{D1Cre}:*Brm*$^{-/-}$ and control mice in the dark-light box test with the time in the lit compartment (left panel) and the latency of first entry in the lit compartment (right panel). Controls n=12, compound mutants n=9. Two-sided Mann-Whitney test for latency p=0.0035. **(c)** Basal locomotor activity during 30 minutes in control (n=15), *Brg1*^{D1Cre} (n=16), *Brm*$^{-/-}$ (n=15) and *Brg1*^{D1Cre}:*Brm*$^{-/-}$ mice (n=14). F(3,56)=3.24; p=0.0287. One-way ANOVA followed by Bonferroni correction control *vs* *Brg1*^{D1Cre}:*Brm*$^{-/-}$ *p=0.0324. Data are represented as mean ± s.e.m. Data are provided in a Source Data file.

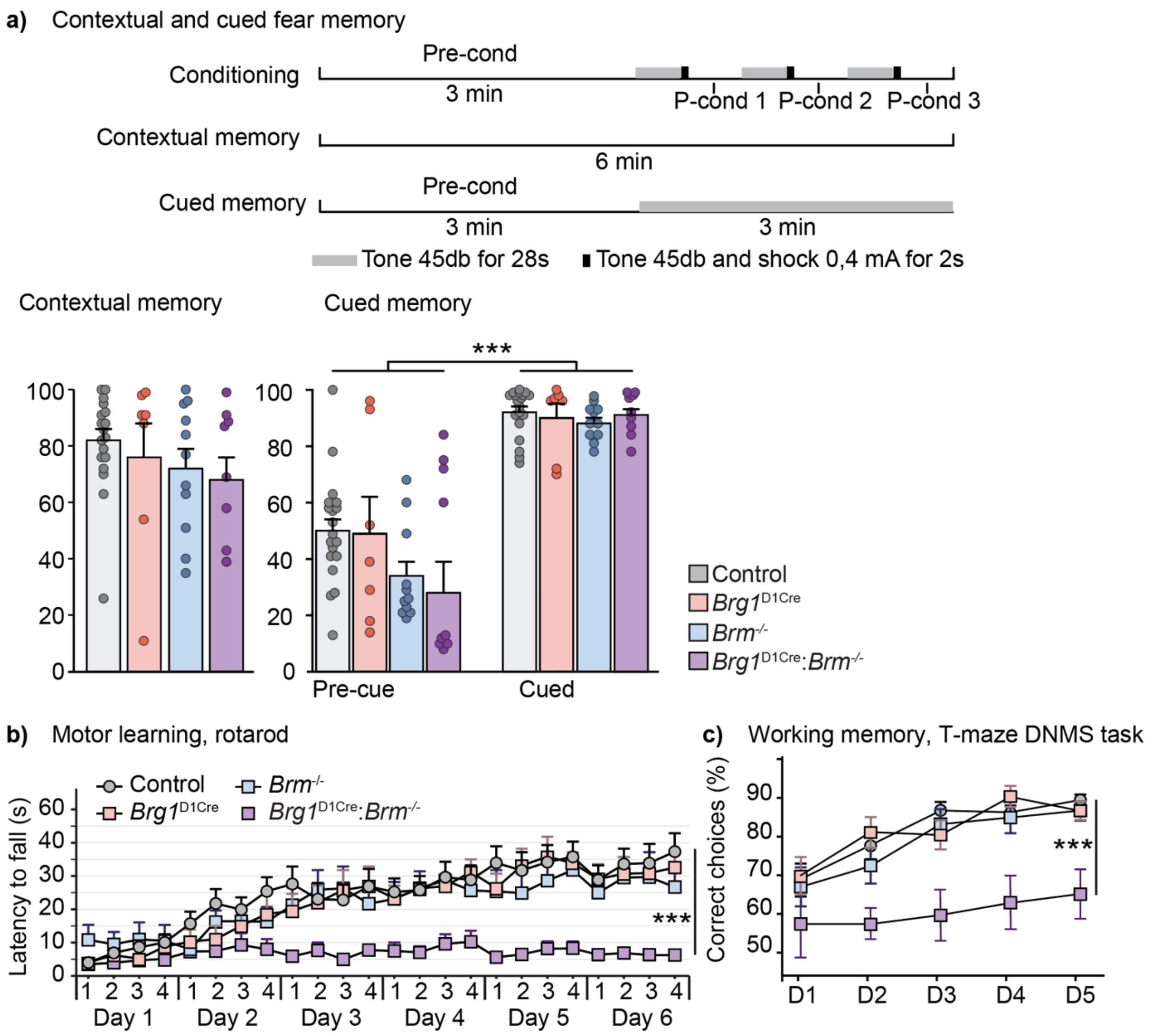

Supplementary Figure 4: Memory and motor coordination in controls, *Brg1*^{D1Cre}, *Brm-/-* and compound mutant mice.

(a) Fear memory in control (n=19), *Brg1*^{D1Cre} mice (n=7), *Brm*$^{-/-}$ (n=11) and *Brg1*^{D1Cre}:*Brm*$^{-/-}$ mice (n=9). Schematic of the fear conditioning protocol with the conditioning (up), the contextual memory (middle) and the cued memory (bottom). Lower left chart, freezing percentage during the contextual fear memory test. One-way ANOVA F(3,42)=0.83; p=0.48. Lower right chart, freezing percentage during the cued fear memory test. Two-way ANOVA with repeated measure, main cue effect F(1,42)=219.7; p<0.0001. **(b)** Motor coordination and learning in control (n=12), *Brg1*^{D1Cre} (n=11), Brm$^{-/-}$ (n=10) and *Brg1*^{D1Cre}:*Brm*$^{-/-}$ mice (n=10). Interaction time x genotype F(23,897)=2.97; p=0.001. Two-way repeated ANOVA with repeated measure followed by Bonferroni correction Day 6-4 control *vs* compound mutant ***p<0.0001. **(c)** T-Maze non-matching to sample working memory task in control (n=22), *Brg1*^{D1Cre} mice (n=13), *Brm*$^{-/-}$ (n=11) and *Brg1*^{D1Cre}:*Brm*$^{-/-}$ mice (n=9). Main days effect F(4,204)=17.3; p<0.0001. Main genotype effect F(3,51)=9.91; p<0.0001. Two-way ANOVA with repeated measure followed by Bonferroni correction Day 5 control *vs* compound mutant ***p<0.0001. Data are represented as mean ± s.e.m. Data are provided in a Source Data file.

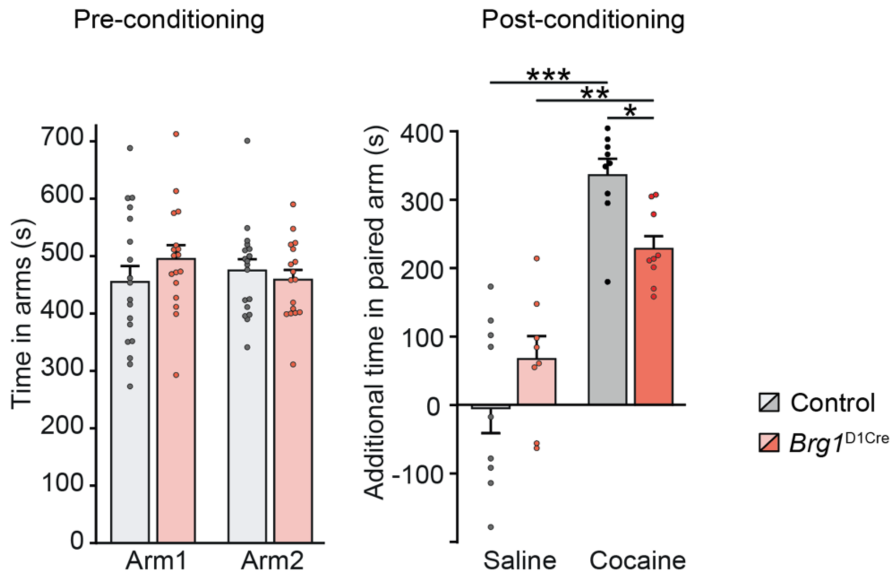

Supplementary Figure 5: Conditioned place preference to cocaine in control and *Brg1*^{D1Cre} mice.

Left chart, time spent in each compartment of the conditioned place preference setup during the preconditioning phase. Control n=18, *Brg1*^{D1Cre} n=17. Right chart, additional time spent in the saline- and cocaine-paired compartment after conditioning in controls and *Brg1*^{D1Cre} mice. Saline- and cocaine-conditioned controls n=9 for each, saline- and cocaine-conditioned *Brg1*^{D1Cre} mice n=8 and n=9 respectively. Main drug effect $F(1,31)=68.8$; $p<0.0001$. Genotype x drug $F(1,31)=8.68$; $p=0.0061$. Two-way ANOVA followed by Bonferroni correction saline vs cocaine ***$p<0.0001$, **$p<0.0016$; cocaine-treated control vs *Brg1*^{D1Cre} *$p=0.0334$. Data are represented as mean ± s.e.m. Data are provided in a Source Data file.

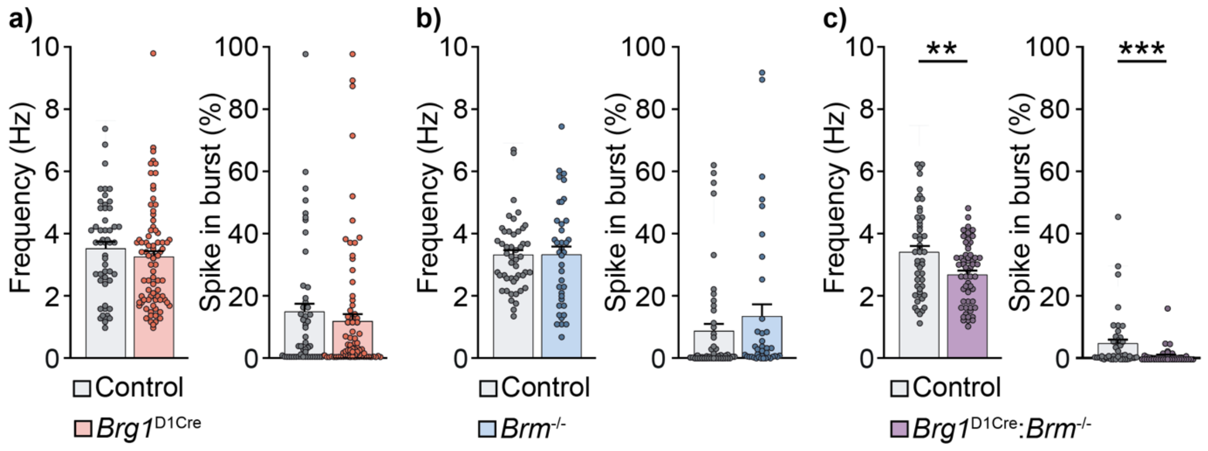

Supplementary Figure 6: Basal spontaneous activity of ventral tegmental area dopamine neurons in control, *Brg1*^{D1Cre}, *Brm*$^{-/-}$ and *Brg1*^{D1Cre}:*Brm*$^{-/-}$ mice.

(a) Firing frequency (left chart) and percentage of spike in burst (right chart) in control (n=49) and BRG1^{D1Cre} mice (n=81) (data from Figure 4a and b). **(b)** Firing frequency (left chart) and percentage of spike in burst (right chart) in control (n=48) and *Brm*$^{-/-}$ mice (n=38). **(c)** Firing frequency (left chart) and percentage of spike in burst (right chart) in control (n=49) and *Brg1*^{D1Cre}:*Brm*$^{-/-}$ mice (n=60). Two-sided T-test (for frequency) and Mann-Whitney test (spike in burst) **p=0.0036, ***p<0.0001. Data are represented as mean ± s.e.m. Data are provided in a Source Data file.

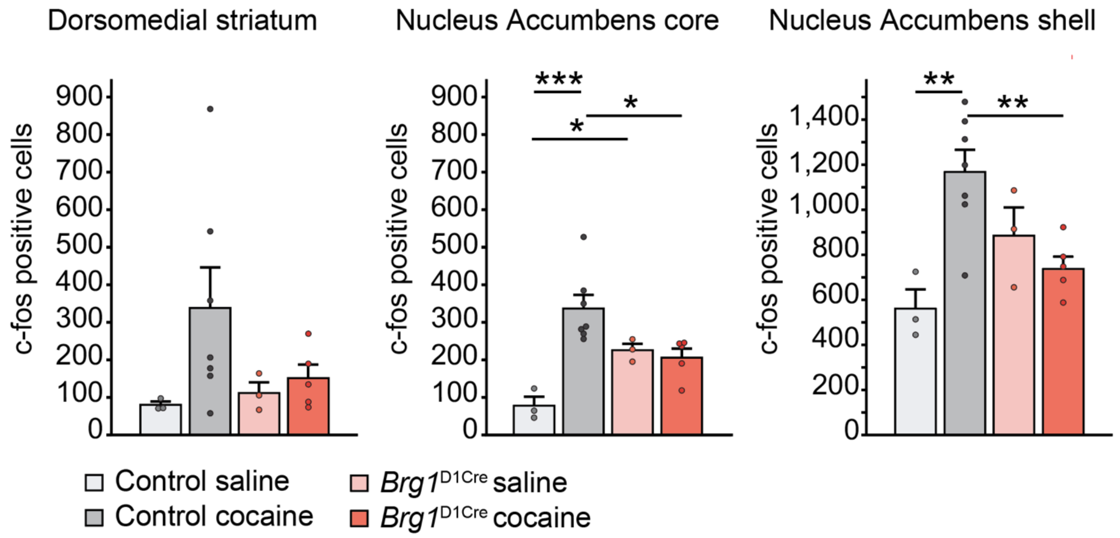

Supplementary Figure 7: *c-fos* gene induction by cocaine in controls and *Brg1*^{D1Cre} mice in the dorsomedial striatum (CPu, left chart), nucleus accumbens core (middle chart) and shell (right chart). Saline-treated control n=3, cocaine-treated control n=7, saline-treated *Brg1*^{D1Cre} n=3, cocaine-treated *Brg1*^{D1Cre} n=5. Two-way ANOVA. NAc core: main drug effect F(1,14)=11.07; p=0.005. Interaction genotype x drug F(1,14)=14.81; p=0.0018. Bonferroni correction saline control vs cocaine control ***p=0.0003, control vs *Brg1*^{D1Cre} saline *p=0.0491; cocaine *p=0.016. NAc shell: main drug effect F(1,14)=5.01; p=0.0419. Interaction genotype x drug F(1,14)=12.6; p=0.0032. Bonferroni correction saline control vs cocaine control **p=0.0017; control vs *Brg1*^{D1Cre} cocaine **p=0.008. Data are represented as mean ± s.e.m. Data are provided in a Source Data file.

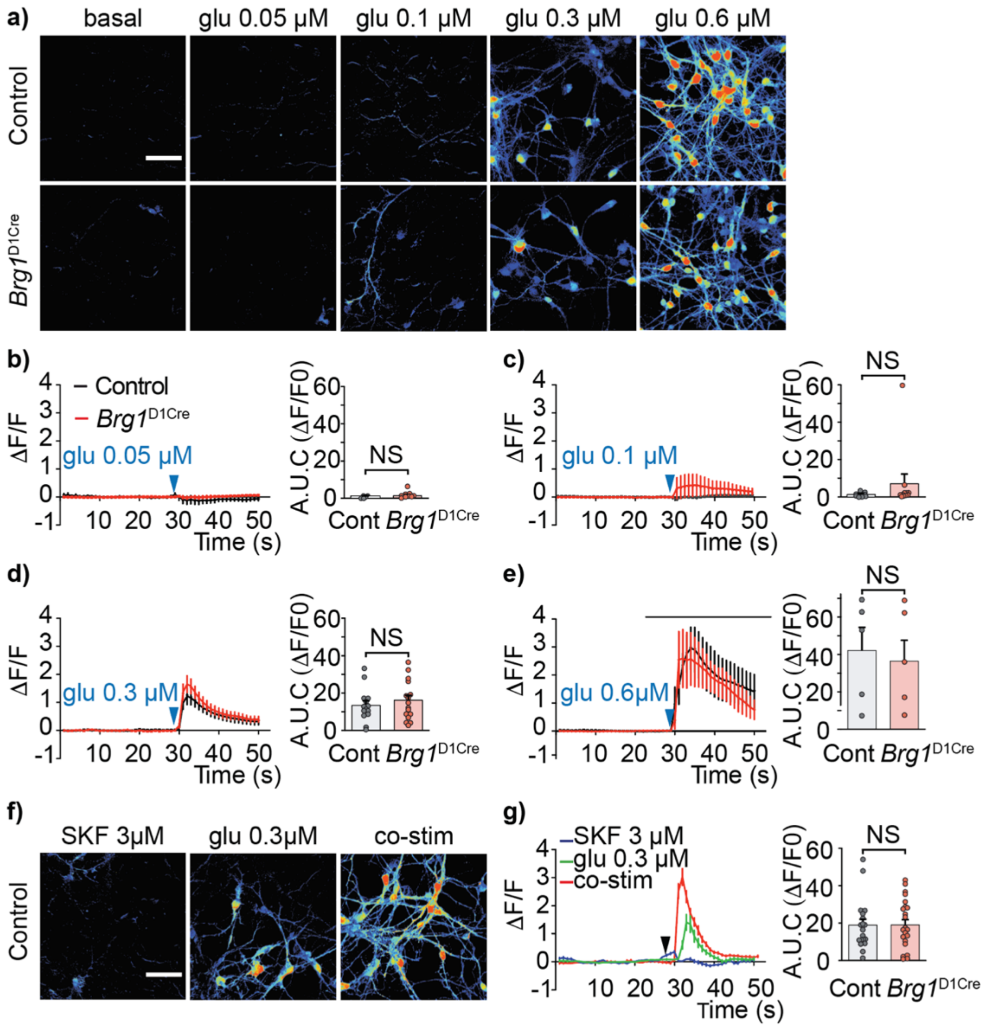

Supplementary Figure 8: Deletion of Brg1 in D1R-MSN preserves glutamate-mediated calcium responses as well as the integration of D1R and glutamate signals.

(a) Representative confocal images of ΔF/F calcium signals obtained at the peak of the responses during glutamate applications at the indicated doses in control (top panels) or *Brg1*^{D1Cre} (bottom panels) cultured striatal neurons. Scale bar : 50 μM. **(b-e)** Left panels: dynamics of calcium signals before (0-30 sec) and during (30 to 50 sec) glutamate applications at the indicated concentrations measured from control (black) or *Brg1*^{D1Cre} (red) neurons. Right panel: quantifications of the corresponding area under the curves (AUC) for control (black bars) and *Brg1*^{D1Cre} (red bars) neurons. Two-way ANOVA with repeated measure, main dose effect $F(4,73)=16.09$ ***$p<0.001$; main genotype effect $F(1,73)=1,01$ $p=0.32$. Controls treated with glutamate 0.05 μM (n=5); 0.1 μM (n=8); 0.3 μM (n=14); 0.6 μM (n=5). *Brg1*^{D1Cre} treated with glutamate 0.05 μM (n=9); 0.1 μM (n=11); 0.3 μM (n=16); 0.6 μM (n=5). **(f)** Representative pictures of the calcium responses elicited by a D1R agonist SKF38393 (3 μM) and glutamate (0.3 μM), applied separately, or together (co-stim) in control neurons. Scale bar : 50 μM. **(g)** Left panel: calcium profiles illustrating the D1R-mediated facilitation of glutamate-mediated calcium transient in control neurons. SKF38393, n=23; glutamate, n=11; costimulation, n=21. Right panel: Quantifications of the AUC of calcium transients triggered by the co-stimulation paradigm in control and *Brg1*^{D1Cre}. Note that the amplitude of the responses elicited by the co-stimulation is similar for both genotypes. Controls treated with SKF 3 μM + glutamate 0.3 μM (n=19). *Brg1*^{D1Cre} treated with SKF 3 μM + glutamate 0.3 μM (n=23). Two-way ANOVA with repeated measure, main drug effect $F(3,96)=9.56$ ***$p<0.001$; main genotype effect $F(1,96)=1.18$ $p=0.28$; NS: not significant. Data are represented as mean ± s.e.m. Data are provided in a Source Data file.

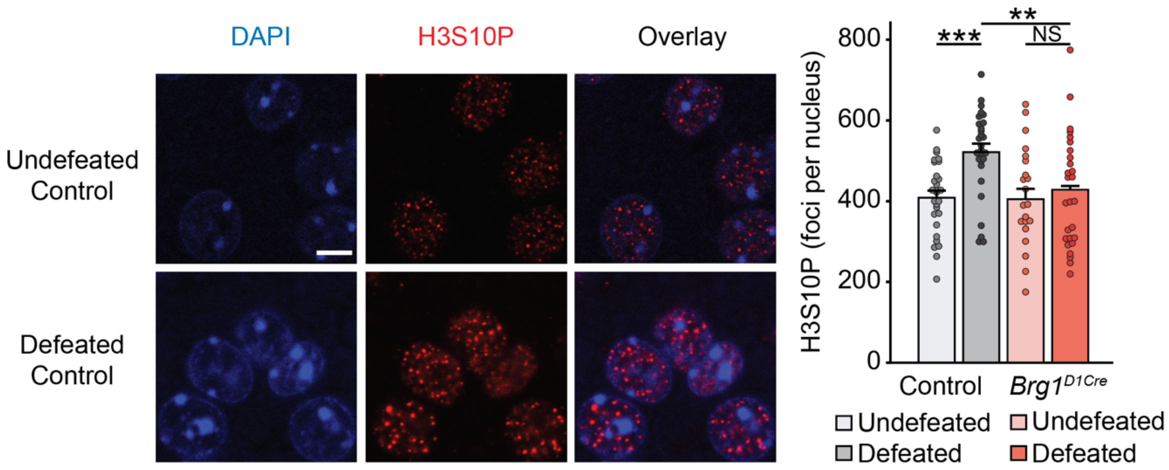

Supplementary Figure 9: Induction of H3S10P after an acute defeat in control and *Brg1*^{D1Cre} mice.

Representative H3S10P staining (red) in the NAc of control mice undefeated and defeated (left panel). Scale bar = 15 µm. Induction of H3S10P by an acute social defeat in control and *Brg1*^{D1Cre} mice (right panel). Undefeated control nuclei n=28 (5 mice), undefeated *Brg1*^{D1Cre} nuclei = 22 (4 mice), defeated control nuclei = 28 (5 mice), defeated *Brg1*^{D1Cre} nuclei = 28 (5 mice). Main genotype effect $F(1,102)=4.42$; $p=0.0038$. Main stress effect $(F1,102)=8.83$; $p=0.0037$. Two-way ANOVA followed by Bonferroni correction control undefeated vs control defeated ***$p=0.0009$, NS: not significant ($p=0.9884$); control defeated vs *Brg1*^{D1Cre} defeated **$p=0.0073$. Data are represented as mean ± s.e.m. Data are provided in a Source Data file.

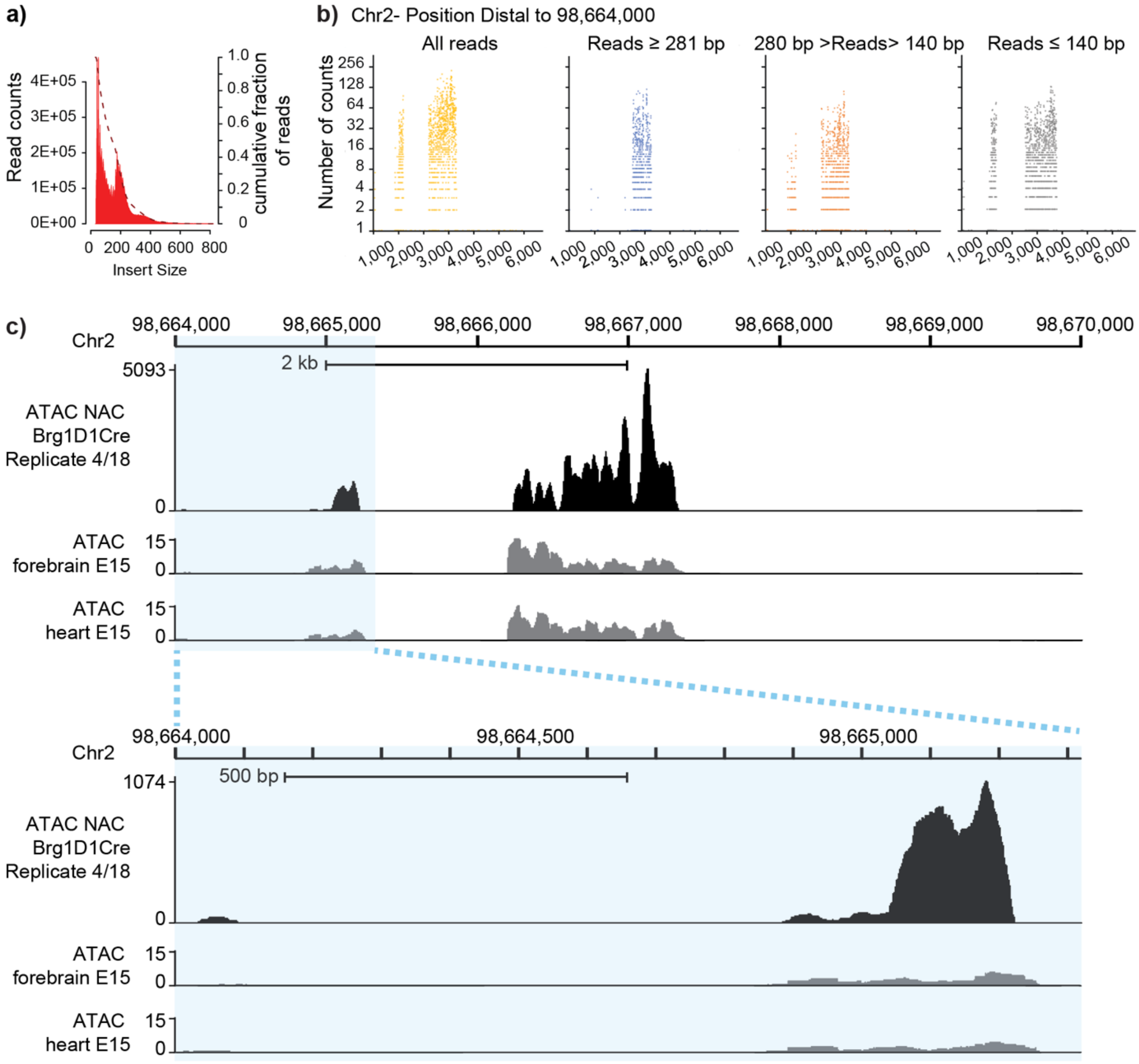

Supplementary Figure 10: Read lengths and DNA accessibility identification.

To compare data from libraries with different ratios of subnucleosomal (<=140 bp), nucleosomal (141-280 bp) and poly-nucleosomal (>=280 bp) read lengths, we first compared the qualitative and quantitative information relative to the accessibility of DNA to the Tn5 transposase by using data from the library corresponding to Mutant *Brg1*^{D1Cre} Replicate 4/18 and a 6-kb region (chr2: 98,664,000-98,670,000) identified by a large read number. (a) Fragment length distributions of the library corresponding to Mutant *Brg1*^{D1Cre} Replicate 4/18. The X-axis indicates read lengths, *i.e.,* the genomic distance between two Tn5 insertion sites as determined by paired-end sequencing of ATAC-seq fragments. The Y-axis indicates the density of the fraction of the reads with the indicated length. **(b)** Reads mapping within the 6-kb genomic segment (chr2: 98,664,000-98,670,000) altogether identified three regions of 70 bp, 200 bp and 1 kb as did reads obtained from P0 mouse forebrain taken as a reference profile at UCSC. **(c)** Only extremities of the subnucleosomal and nucleosomal read subsets identified the three regions. The poly-nucleosomal read subset missed the 70-bp region and identified only part of the 1 kb one. The subnucleosomal read subset displayed the more complete picture of DNA accessibility. Data are provided in a Source Data file.

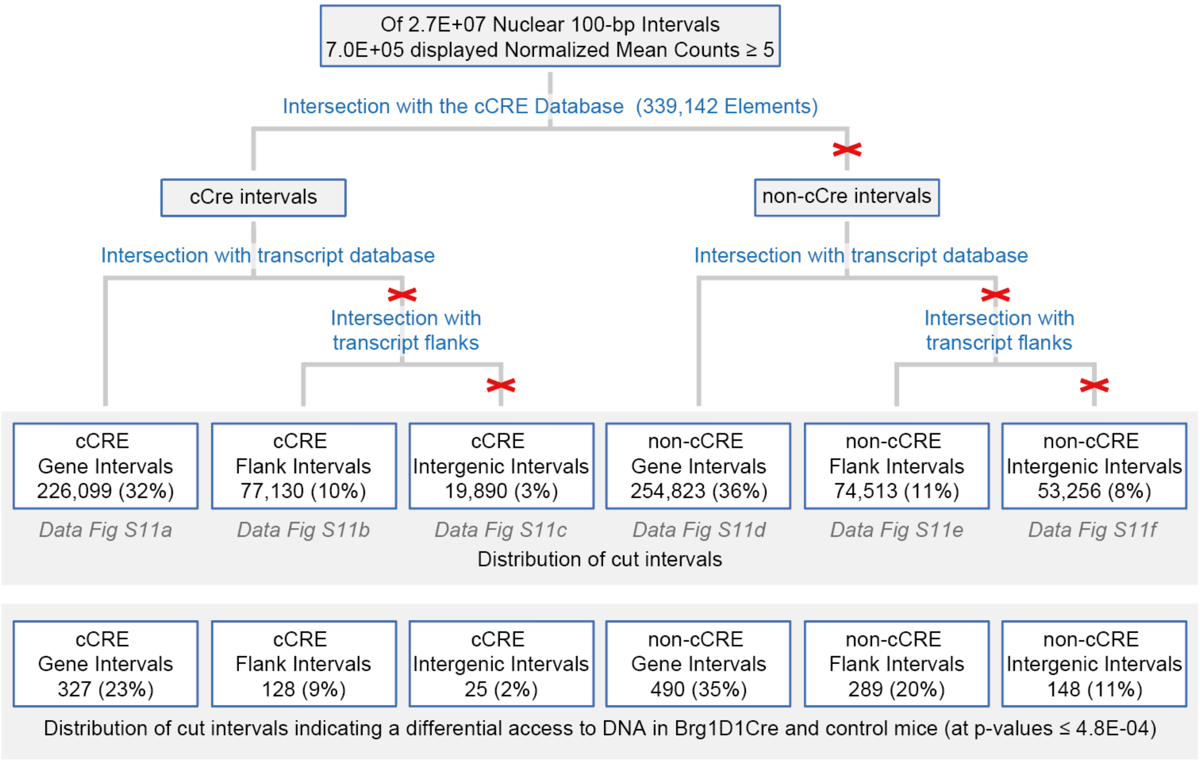

Supplementary Figure 11. Chart of the analysis of genome accessibility.

Genome accessibility was performed on 7 *Brg1*^{D1Cre} and 6 control mice. The mouse genome was binned into 2.7E+7 intervals of 100 bp and the 7.1E+05 (2.4%) accessible intervals identified by a mean of 5 or more read extremities were analyzed for their overlap with cCREs, genes and gene-flanking sequences (corresponding accompanying Data files are indicated in grey letters). Data are provided in a Source Data file.

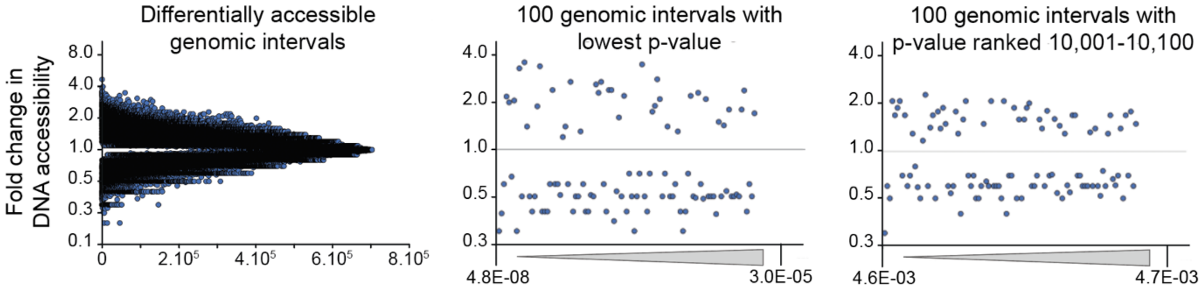

Supplementary Figure 12. Glances at fold-changes in DNA accessibility between mutant and control chromatins.

Fold-changes in accessibility are pictured for all the differentially accessible genomic intervals (left panel), the 100 ones with lowest p-values ranging from 4.8E-08 to 3.0E-05 (middle panel), and for 100 higher p-values ranging from 4.6E-03 to 4.7E-03 (right panel), two-sided Mann-Whitney test. See accompanying Data file. Data are provided in a Source Data file.

Antibody	Dilution	Specie	Type	Provider	Catalog	Lot	Reference	Specificity
**BRG1**	1/1,000	Mouse	Monoclonal (clone G-7)	Santa Cruz	sc-17796	H1314	Present article	Absence of immunostaining in *Brg1* ^{D1Cre} mice MSNs
**BRM**	1/500	Rabbit	Polyclonal	Abcam	ab15597	GR49552-3	Present article	Absence of immunostaining in *Brm* $^{-/-}$ mice
**c-Fos**	1/1,000	Mouse	Monoclonal (clone E-8)	Santa Cruz	sc-166940	H2420	Zhang et al. 2006[62]	Absence in c-Fos^{D1Cre} mice
**EGR1**	1/500	Rabbit	Monoclonal clone EPR5014(2)	Abcam	ab133695	n.a.	Liu et al. 2018[75]	Hippocampal induction with spatial learning
**GR**	1/1,000	Rabbit	Polyclonal	Santa Cruz	M-20 sc-1004	B0315	Ambroggi et al. 2009[8]	Absence in neurons of GR gene mutant mice
**H3K9me3**	1/400	Rabbit	Polyclonal	Abcam	ab8898	GR3302452-1	Sanchez et al. 2019[76]	Colocalize with a biosensor probing for H3K9me3 in cell culture
**H3S10P**	1/1,000	Rabbit	Polyclonal	Millipore	06-570	JBC1903648	Salery et al. 2017[42]	Neuronal expression induced by cocaine treatment
**HP1**	1/1,000	Rabbit	Monoclonal clone EPR5777	Abcam	ab109028	GR44062-29	Strom et al. 2021[77]	Absence of immunostaining after degradation of HP1 in cell culture
**Lamin B1**	1/1,000	Rabbit	Polyclonal	Abcam	ab16048	GR297590-3	Nmezi et al. 2019[78]	Absence in LaminB1 knockout mice
**NeuN**	1/200	Mouse	Monoclonal clone A60	Millipore	MAB377	NG1876252	Jacko et al. 2018 [79]	Absence in NeuN (Rbfox3) gene mutant mice (western and immunohistochemistry)
**pERK**	1/400	Rabbit	Monoclonal clone D13.14.4E	Cell Signaling Technology	#4370	28	Contesse et al. 2021[80]	Induction by stress and blocked by glutamate afferent silencing

Supplementary Table 1: List of primary antibodies.

Mouse		Reads ≤140 bp subnucleosomal (number)	140 < reads > 281 bp nucleosomal (number)	281 bp ≤ reads poly-nucleosomal (number)	Total reads (number)	Reads ≤140 bp subnucleosomal (%)	140 < reads > 281 bp nucleosomal (%)	281 bp ≤ reads poly-nucleosomal (%)	Total reads (%)
Control	1/1	3,2E+07	6,2E+06	2,0E+05	3,8E+07	0,83	0,16	0,01	1,00
	2/3	2,1E+07	6,1E+06	1,4E+05	2,7E+07	0,77	0,22	0,01	1,00
	3/6	2,5E+07	3,0E+07	2,9E+06	5,8E+07	0,43	0,52	0,05	1,00
	4/7	2,8E+07	5,9E+06	2,2E+05	3,4E+07	0,82	0,17	0,01	1,00
	5/22	9,6E+05	1,7E+06	1,1E+06	3,8E+06	0,26	0,45	0,29	1,00
	6/23	1,3E+06	4,1E+06	3,0E+06	8,4E+06	0,15	0,49	0,36	1,00
	7/25	1,6E+07	9,6E+06	1,7E+06	2,7E+07	0,59	0,35	0,06	1,00
	8/28	2,8E+07	6,5E+06	5,5E+05	3,5E+07	0,80	0,19	0,02	1,00
Brg1D1Cre	1/2	1,7E+07	1,5E+07	1,2E+06	3,3E+07	0,51	0,45	0,04	1,00
	2/4	1,9E+07	7,6E+05	6,4E+04	2,0E+07	0,96	0,04	0,00	1,00
	3/5	3,3E+07	1,5E+07	6,0E+05	4,9E+07	0,68	0,31	0,01	1,00
	4/18	3,5E+07	2,7E+07	6,8E+06	6,9E+07	0,51	0,39	0,10	1,00
	5/20	2,5E+07	9,3E+06	9,6E+05	3,5E+07	0,71	0,26	0,03	1,00
	6/21	2,2E+06	1,0E+07	2,9E+06	1,5E+07	0,15	0,66	0,19	1,00
	7/24	1,1E+06	3,9E+06	2,2E+06	7,2E+06	0,15	0,54	0,31	1,00
	8/26	1,9E+07	1,0E+07	1,5E+06	3,1E+07	0,62	0,33	0,05	1,00
	9/27	2,9E+07	5,6E+06	6,3E+05	3,5E+07	0,82	0,16	0,02	1,00

Supplementary Table 2: Read length distribution.

We constructed 17 libraries encompassing 3.8E+6 to 6.9E+7 reads. Reads were aligned to the mouse mm10 reference genome, mitochondrial and nuclear PCR duplicate reads, removed, and proper pairs (excluding those mapped to unmapped contigs), selected. Unique nuclear reads displayed different ratios of subnucleosomal (≤140 bp), nucleosomal (141-280 bp) or poly-nucleosomal (≥281 bp) lengths that were compared for their qualitative and quantitative information. Control animals 5/22 and 6/23 and $Brg1^{D1Cre}$ animals 6/21 and 7/24 were excluded of our analysis because of their low number (9.6E+05 to 2.2E+06 reads) of reads of subnucleosomal length when compared to the others (1.7E+07 to 3.3E+07 reads).

	Term	Count	%	p-value	Benjamini
KEGG Pathway	Long-term depression	9	1,2	1,2E-03	2,7E-01
	Morphine addiction	10	1,4	4,9E-03	3,1E-01
	Axon guidance	12	1,6	5,2E-03	3,1E-01
	Hippo signaling pathway	13	1,8	6,3E-03	3,1E-01
	Regulation of actin cytoskeleton	16	2,2	7,4E-03	3,1E-01
	Pancreatic secretion	10	1,4	7,9E-03	3,1E-01
Tissue	Brain	353	48,0	2,0E-09	3,6E-07
	Brain Cortex	50	6,8	1,8E-08	1,6E-06
	Squamous Cell Carcinoma	5	0,7	1,3E-05	8,0E-04
	Fetal Brain	24	3,3	7,8E-04	3,0E-02
	Eyeball	22	3,0	8,4E-04	3,0E-02
	Cerebellum	79	10,7	1,1E-03	3,2E-02
	Diencephalon	23	3,1	2,6E-03	6,0E-02
	Embryonic tail	25	3,4	3,2E-03	6,0E-02
	Eye	79	10,7	3,4E-03	6,0E-02
	Adult Thymus	6	0,8	3,4E-03	6,0E-02
	Adrenal Gland	7	1,0	6,6E-03	1,1E-01

Supplementary Table 3: Gene Ontology analyses.

The 1,273 DNA intervals of lowest p-values (4.8E-08 to 4.8E-04), corresponding to 736 genes. were analyzed for gene ontologies. The number of genes involved, and the corresponding percentage are indicated, as well as the associated p-value (two-sided Fischer exact test) and adjusted p-value (Benjamini correction).
