# Supplementary material for: SWI/SNF chromatin remodeler complex within the reward pathway is required for behavioral adaptations to stress
Source: Nat Commun. 2022 Apr 4;13:1807. doi: 10.1038/s41467-022-29380-8 (PMC8980038; doi:10.1038/s41467-022-29380-8)
Supplement: Supplementary file 3 — Reporting summary [file 41467_2022_29380_MOESM3_ESM.pdf]

nature research

Corresponding author(s): François Tronche

Last updated by author(s): Jan 6, 2022

Reporting Summary

Nature Research wishes to improve the reproducibility of the work that we publish. This form provides structure for consistency and transparency in reporting. For further information on Nature Research policies, see Authors & Referees and the Editorial Policy Checklist .

Statistics

For all statistical analyses, confirm that the following items are present in the figure legend, table legend, main text, or Methods section.

n/a	Confirmed	
☐	☒	The exact sample size (n) for each experimental group/condition, given as a discrete number and unit of measurement
☐	☒	A statement on whether measurements were taken from distinct samples or whether the same sample was measured repeatedly
☐	☒	The statistical test(s) used AND whether they are one- or two-sided<br>*Only common tests should be described solely by name; describe more complex techniques in the Methods section.*
☒	☐	A description of all covariates tested
☐	☒	A description of any assumptions or corrections, such as tests of normality and adjustment for multiple comparisons
☐	☒	A full description of the statistical parameters including central tendency (e.g. means) or other basic estimates (e.g. regression coefficient) AND variation (e.g. standard deviation) or associated estimates of uncertainty (e.g. confidence intervals)
☐	☒	For null hypothesis testing, the test statistic (e.g. F, t, r) with confidence intervals, effect sizes, degrees of freedom and P value noted<br>*Give P values as exact values whenever suitable.*
☒	☐	For Bayesian analysis, information on the choice of priors and Markov chain Monte Carlo settings
☒	☐	For hierarchical and complex designs, identification of the appropriate level for tests and full reporting of outcomes
☒	☐	Estimates of effect sizes (e.g. Cohen's d, Pearson's r), indicating how they were calculated

Our web collection on statistics for biologists contains articles on many of the points above.

Software and code

Policy information about availability of computer code

Data collection	For collection of behavioral data we used Ethovision XT software (Noldus, version 14.0)<br>For confocal images collection, we used Leica-Application-Suite Advanced Fluorescence software (version 2.7.3.9723)
Data analysis	We used open source data analyses interfaces (ATACSeq) of the usegalaxy platform (https://usegalaxy.org), including Bowtie2 (Version 2.4.2), Samtools (Version 1.11) and Picard Tools (Version 2.18.2).<br>We used Fili open access software for images analyses (version 2.1.0/1.53c).

For manuscripts utilizing custom algorithms or software that are central to the research but not yet described in published literature, software must be made available to editors/reviewers. We strongly encourage code deposition in a community repository (e.g. GitHub). See the Nature Research guidelines for submitting code & software for further information.

Data

Policy information about availability of data

All manuscripts must include a data availability statement. This statement should provide the following information, where applicable:
- Accession codes, unique identifiers, or web links for publicly available datasets
- A list of figures that have associated raw data
- A description of any restrictions on data availability

The ATAC-Seq data generated in this study have been deposited in the SRA database under accession code PRJNA751751 (https://www.ncbi.nlm.nih.gov/sra/?term=PRJNA751751). For analyses, we used publicly available datasets, including mouse mm10 reference genome (https://www.ncbi.nlm.nih.gov/assembly/GCF_000001635.20/), GR ChIPseq study from cultured mouse embryonic fibroblasts 56 (https://www.ncbi.nlm.nih.gov/geo/query/acc.cgi?acc=GSE126655), and ATAC-seq profiles obtained from mouse forebrain E15 (https://www.encodeproject.org/experiments/ENCSR976LWP/) and P0 (https://www.encodeproject.org/experiments/ENCSR310MLB/). Figures 1 to 7 and Supplementary Figures 1 to 12 have associated raw data present in the provided Dataset files. There is no restriction on data availability.

Field-specific reporting

Please select the one below that is the best fit for your research. If you are not sure, read the appropriate sections before making your selection.

☒ Life sciences ☐ Behavioural & social sciences ☐ Ecological, evolutionary & environmental sciences

For a reference copy of the document with all sections, see nature.com/documents/nr-reporting-summary-flat.pdf

Life sciences study design

All studies must disclose on these points even when the disclosure is negative.

Sample size

Sample size for histology, behavioral experiments, electrophysiological recordings, Ca2+ imaging, Erk pathway (including immediate early genes induction, pERK, H3S10P, HP1, H3K9me3) were therefore determined based on previous experience (Barik et al. Biol. Psy 2010; Barik et al. Science 2013, Cahill et al. Mol Psy 2014, Salery et al. Biol Psy 2017).
Sample size for ATAC-Seq was estimated based on Wenderski et al. (PNAS 2020) analysing the consequences in neurons of ACTL6B gene inactivation, encoding a Brg1 associated factor (BAF53b), in which they analyzed 5 to 7 samples.

Data exclusions

One undefeated control was excluded from the analysis of Egr1 induction (Figure 6a), it was above the mean plus two times the standard error for all structures measured. Some experimental animals were excluded before analysis due to problems during experiment, such as failures in video recording (social interaction, Figure 2a), missed dissection of the adrenals (Figure S3), failure of habituation in locomotor activity chambers (Figure 3), and damaged tissue (Figure 6b).

Replication

GR-SWI/SNF co-immunoprecipitations, acute GR-BRG1 co-localizations, NeuN staining quantifications, social avoidance and anxiety tasks following repeated social defeats in Brg1D1Cre and Brm-/- , electrophysiological recordings of dopamine neurons, Ca2+ imaging, quantification of heterochromatin amount in DAPI stainings, have been performed in several independent experiments yielding the same results. DAPI stainings have been confirmed with H3K9me3 and HP1 stainings. Differences in induction of c-Fos gene expression between Brg1D1Cre and control mice have been examined upon two different conditions (cocaine and stress responses) giving similar results.
Cocaine sensitization has been done once. The effects of the mutations were clear and coherent with the results obtained with Brg1D1Cre mice in conditioned place preference to cocaine as well as in line with experiments showing decreased sensitization to morphine in that model. Rotarod data come from a single cohort. The results show very low variability and match the locomotor activity measured in figure S3c. Social aversion following repeated defeats in the compound mutants and in NAcc inactivated model result from a single experiment, done upon reviewers' request. The data follow our findings on Brg1D1Cre and Brm-/- models. Finally, fear memory and T-maze in the Brm-/- and compound mutants have been done once but showed very little variability with a cohort size between 9 and 11 animals.
We measured once the nuclear shape abnormalities as well as the BRM and BRG1 levels in respective mutants as these results were in line with already published data (Imbalzano et al. 2013, and Reyes et al.1998, respectively). ATACseq sequencing experiments were performed within standard criteria for this approach. Each sample used 6 to 7 biological replicates of NAc. The regions of accessible chromatin in each biological replicate match the region of accessible chromatin identified in similar experiments. Furthermore, the differentially accessible regions between control and mutant NAcc are enriched in brain specific genes.
This has been added to the Methods section.

Randomization

Before behavioral procedures, groups of animals (mutants, controls, treatments) were formed paying attention for balance in age and cage origin. During behavioral session, genotypes and treatments were alternated to avoid bias due to the experimental design.
For histology each well included a slice from each genotype/condition. For molecular biology, nuclear preparations were performed on two controls and two mutant each day, alterning between genotypes.
For all experiments, but especially for behavioral ones, we paid attention to possible effects of circadian rythm. If requiring several days, experiments were run within the same daily 2-3 hours windows.

Blinding

Data collections and analysis were performed blindly.

Reporting for specific materials, systems and methods

We require information from authors about some types of materials, experimental systems and methods used in many studies. Here, indicate whether each material, system or method listed is relevant to your study. If you are not sure if a list item applies to your research, read the appropriate section before selecting a response.

Materials & experimental systems

n/a	Involved in the study
☐	☒ Antibodies
☒	☐ Eukaryotic cell lines
☒	☐ Palaeontology
☐	☒ Animals and other organisms
☒	☐ Human research participants
☒	☐ Clinical data

Methods

n/a	Involved in the study
☒	☐ ChIP-seq
☒	☐ Flow cytometry
☒	☐ MRI-based neuroimaging

Antibodies

Antibodies used

Primary antibodies :
Anti-BRG1 (Mouse monoclonal, clone G-7, Santa Cruz, Catalog sc-17796 Lot H1314).
Anti-BRM (Rabbit polyclonal, Abcam, Catalog ab15597 #GR49552-3).
Anti c-FOS (Mouse monoclonal, clone E-8, Santa Cruz, Catalog sc-166940, Lot H2420).
Anti-EGR1 (Rabbit monoclonal, clone EPR5014(2), Abcam, Catalog ab133695).
Anti-GR (Rabbit polyclonal, Santa Cruz M20, Catalog sc-1004, Lot B0315).
Anti-H3K9me3 (Rabbit polyclonal, Abcam, Catalog ab8898, Lot GR3302452-1).
Anti-HP1 (Rabbit monoclonal, clone EPR5777, Abcam, Catalog ab109028, Lot GR44062-29).
Anti-H3S10P (Rabbit polyclonal, Millipore, Catalog 06-570, Lot JBC1903648).
Anti-Lamin B1 (Rabbit polyclonal, Abcam, Catalog ab16048, Lot GR297590-3).
Anti-NeuN (Mouse monoclonal, clone A60, Millipore, MAB377, Lot NG1876252).
Anti-pERK (Rabbit, monoclonal, clone D13.14.4E Cell Signaling Technology, Catalog 4370, Lot 28).
Secondary antibodies:
anti-rabbit peroxidase-conjugated (Donkey polyclonal, Jackson Laboratories, Catalog 711-035-152, Lot 94250).
anti-rabbit Alexa488 (Goat polyclonal, Invitrogen, Catalog A11008).
anti-mouse Alexa488 (Goat polyclonal, Life Technology, Catalog A110029, Lot 1531669).
anti-rabbit CY3 (Goat polyclonal, Invitrogen, Catalog A10520).
anti-mouse CY3 (Goat polyclonal, Invitrogen, Catalog A10521).

Validation

Anti-BRG1 validated by the absence of immunostaining in Brg1D1Cre mice MSNs (this manuscript).
Anti-BRM validated by the absence of immunostaining in Brm-/-1 mice MSNs (this manuscript).
Anti c-FOS. validated by the absence of immunostaining in c-FosD1Cre mice (Zhang et al. 2006, cited). Validation by the manufacturer : https://www.scbt.com/scbt/product/c-fos-antibody-e-8
Anti-EGR1. Validation by the manufacturer : https://www.abcam.com/egr1-antibody-epr50142-ab133695.html. Validation by hippocampal induction with spatial learning (Liu et al. 2018, cited).
Anti-GR. We validated this antibody by the absence of immunostaings in neurons of GR gene mutant mice GR mutant mice. See for instance Barik et al. 2013.
Anti-H3K9me3, validated by colocalization with a biosensor probing for H3K9me3 in cell culture (Sanchez et al. 2019, cited).
Anti-H3S10P. Validated by induced neuronal expression following cocaine treatment (Salry et al. 2017, cited).
Anti HP1, Validated by absence of immunostaining after degradation of HP1 in cell culture (Strom et al. 2021, cited).
Anti-Lamin B1, validated by absence of signal in LaminB1 knockout mice (Nmezi. et al. 2019, cited). Validation by the manufacturer : https://www.abcam.com/lamin-b1-antibody-nuclear-envelope-marker-ab16048.html.
Anti-NeuN. Validated by absence of signal in NeuN (Rbfox3) gene mutant mice (western and immunohistochemistry, Jacko et al. 2018, cited). Validation by the manufacturer : https://www.merckmillipore.com/FR/fr/product/Anti-NeuN-Antibody-clone-A60,MM_NF-MAB377
Anti-pERK. Validated by induction following stress, blocked by glutamate afferent silencing (Contsse et al. 2021, cited). Validation by the manufacturer : https://www.cellsignal.com/products/primary-antibodies/phospho-p44-42-mapk-erk1-2-thr202-tyr204-d13-14-4e-xp-rabbit-mab/4370

Animals and other organisms

Policy information about studies involving animals; ARRIVE guidelines recommended for reporting animal research

Laboratory animals

Experiments were performed on Mus musculus. Experimental animals were 2 to 4 month old maintained on a mixed 129SVeV and C57BL/6J background. In addition, in experiments we used C57BL/6 (2 to 4 month old) males for social interactions in the three chamber test and CD1 males (6 to 12 month old) for the repeated social defeat protocol.

Wild animals

No wild animals were used in the study.

Field-collected samples	No field collected samples were used in the study.
Ethics oversight	The study protocol was approved by the Comité d'éthique pour l'expérimentation animale Charles Darwin (05, Paris). Avis n° #7074 2016091623299400 v5

Note that full information on the approval of the study protocol must also be provided in the manuscript.
